# Comparison of gut microbiome composition in colonic biopsies, endoscopically-collected and at-home-collected stool samples

**DOI:** 10.3389/fmicb.2023.1148097

**Published:** 2023-06-01

**Authors:** Christina Nowicki, Lucille Ray, Philip Engen, Andrea Madrigrano, Thomas Witt, Thomas Lad, Melody Cobleigh, Ece A. Mutlu

**Affiliations:** ^1^Department of Internal Medicine, Division of Digestive Diseases and Nutrition, Rush University Medical Center, Chicago, IL, United States; ^2^Department of Medicine, Division of Gastroenterology and Hepatology, University of Illinois at Chicago, Chicago, IL, United States; ^3^Rush Center for Integrated Microbiome and Chronobiology Research, Rush University Medical Center, Chicago, IL, United States; ^4^Department of Surgery, Rush Medical College, Rush University Medical Center, Chicago, IL, United States; ^5^Department of Oncology, Cook County Health and Hospital Systems, Chicago, IL, United States; ^6^Department of Internal Medicine, Division of Hematology, Oncology, and Cell Therapy, Rush University Medical Center, Chicago, IL, United States

**Keywords:** human microbiome, 16S rRNA gene, intestinal microbiome, mucosal microbiome, lumen, mucosal biopsy, bacteria, microbiome sampling

## Abstract

**Aim:**

The goal of this study is to compare microbiome composition in three different sample types in women, namely stool brought from home vs. solid stool samples obtained at the time of an unprepped sigmoidoscopy vs. biopsies of the colonic mucosa at the time of an unprepped sigmoidoscopy, using alpha- and beta-diversity metrics following bacterial 16S rRNA sequencing. The findings may have relevance to health and disease states in which bacterial metabolism has a significant impact on molecules/metabolites that are recirculated between the gut lumen and mucosa and systemic circulation, such as estrogens (as in breast cancer) or bile acids.

**Methods:**

Concomitant at-home-collected stool, endoscopically-collected stool, and colonic biopsy samples were collected from 48 subjects (24 breast cancer, 24 control.) After 16S rRNA sequencing, an amplicon sequence variant (ASV) based approach was used to analyze the data. Alpha diversity metrics (Chao1, Pielou’s Evenness, Faith PD, Shannon, and Simpson) and beta diversity metrics (Bray-Curtis, Weighted and Unweighted Unifrac) were calculated. LEfSe was used to analyze differences in the abundance of various taxa between sample types.

**Results:**

Alpha and beta diversity metrics were significantly different between the three sample types. Biopsy samples were different than stool samples in all metrics. The highest variation in microbiome diversity was noted in the colonic biopsy samples. At-home and endoscopically-collected stool showed more similarities in count-based and weighted beta diversity metrics. There were significant differences in rare taxa and phylogenetically-diverse taxa between the two types of stool samples. Generally, there were higher levels of Proteobacteria in biopsy samples, with significantly more Actinobacteria and Firmicutes in stool (all *p* < 0.001, q-value < 0.05). Overall, there was a significantly higher relative abundance of *Lachnospiraceae* and *Ruminococcaceae* in stool samples (at-home collected and endoscopically-collected) and higher abundances of *Tisserellaceae* in biopsy samples (all *p* < 0.001, q-value < 0.05).

**Conclusion:**

Our data shows that different sampling methods can impact results when looking at the composition of the gut microbiome using ASV-based approaches.

## 1. Introduction

The gut microbiome consists of a large number of microorganisms within the gut lumen as well as the mucosal surface of the digestive tract. To date, many studies have examined gut microbiome composition and function in both normal and disease settings (Schmidt et al., 2018; Adak and Khan, 2019). The current body of work has demonstrated differences between the gut lumen samples (such as feces) and gut-mucosa associated samples (obtained through biopsies of the intestinal tract; Zoetendal et al., 2002; Carroll et al., 2010; Gillevet et al., 2010; Momozawa et al., 2011; Rangel et al., 2015; Ringel et al., 2015; Shobar et al., 2016; Tap et al., 2017; Altomare et al., 2019; Kim et al., 2021; Jiao et al., 2022). In one study by Flynn et al., significant differences between microbiome composition in the stool and mucosal biopsy samples were seen in several different intestinal sites (Flynn et al., 2018). Such differences between the gut mucosa-associated samples (i.e., biopsies of the mucosa) and luminal samples (i.e., fecal samples, rectal swabs, luminal washings at the time of colonoscopy, etc.) can be expected due to significant differences between these two microenvironments. For example, the gut lumen is known to be largely anaerobic, whereas there is diffusion of oxygen onto the mucosal surface with a distinct oxygen gradient (Albenberg et al., 2014). The mucosal surface is also characterized with large amounts of mucus as well as immune cells and their products (e.g., IgA from B cells and reactive oxygen species from innate immune cells which can affect bacterial composition and function).

Despite these differences, few studies have examined whether it is best to study mucosal samples vs. that of feces in a given disease: for example, in inflammatory bowel disease in which there is significantly increased immune cells within the gut mucosa, there is a loss of difference between gut microbiome composition in the mucosal and luminal samples (Gillevet et al., 2010). However, in many other disease states, it is unclear whether differences in the microbiome remain with mucosal or luminal sampling methods, and which samples (mucosal vs. luminal vs. both sample types) are necessary to understand the effects of the microbiome on disease pathogenesis or course. Breast cancer (BC) is one such disease state. It has been postulated that in BC, gut microbiome composition and function may differ (Bobin-Dubigeon et al., 2021; Ma et al., 2022) and could affect the enterohepatic recirculation of estrogens and thereby have an impact on disease pathogenesis (Plottel and Blaser, 2011). Estrogen reabsorption requires bacterial deconjugation of estrogen from sulfate- or glucuronyl-residues (Birnbaum et al., 2016). It is currently unknown whether this process occurs mostly within the lumen or at the mucosal surface, which exact taxa contribute to the majority of this process, and whether it is the rare taxa or the most abundant ones. As such, it would be important to understand how different luminal vs. mucosal samples are in the context of a subject population relevant to BC (i.e., older peri/post-menopausal women with age > 40 who are target populations in need of BC screening), and whether obtaining mucosal biopsies from the intestine of women (which are expected to be more indicative of bacterial metabolism at the surface of the intestinal tract) can give different and/or complementary information to that obtained from fecal samples.

Unlike fecal samples, mucosal biopsies (expected to be reflective of the mucosa-associated gut microbiome) are hard to obtain, requiring an invasive method, such as endoscopy, sigmoidoscopy, or colonoscopy. However, mucosal biopsies have the benefit of being able to be preserved immediately after collection by flash-freezing methods in liquid nitrogen at the endoscopy lab. Published studies note differences in mucosal vs. stool samples, and also note that bowel preparation can change microbiota composition (Nagata et al., 2019; Powles et al., 2022). Some studies have investigated the difference between endoscopically-collected mucosal and lavage (luminal) samples (Kim et al., 2021; Miyauchi et al., 2022). To date, only one study has specifically investigated differences in the microbiome in the endoscopically-collected mucosal and fecal samples (Shobar et al., 2016). However, this study included a colon prep prior to procedure.

In highly accessible fecal samples, the amount of microbiome sampled is large relative to that of mucosal biopsies, rendering stool samples suitable for metagenomics and meta-transcriptomics applications. One potential disadvantage is that there can often be variations in sampling procedures completed at-home by an individual subject enrolled in a research study, as well as delays in time between when a subject defecates at-home and then collects the stool and when they are properly stored in a research lab. Theoretically, changes in collection methods (in aerobic collection containers vs. anaerobic containers, or in various media vs. no media) as well as storage conditions, such as temperature and oxygen concentration, can also potentially lead to changes in the microbiome composition or function, through either bacterial death or growth during adaption to a new/different environment outside of the human body. Some studies have shown the short-term stability of stool samples by placing freshly expressed stool in an ambient environment at room temperature without refrigeration (Choo et al., 2015). However, there is little to no published data on how microbiome composition and/or function can be affected in fecal samples collected endoscopically with CO2 insufflation during an unprepped endoscopic procedure vs. those brought in from home by subjects.

Thus, the goal of this study is to compare microbiome composition in three different sample types in women, namely stool brought from home vs. solid stool samples obtained at the time of an unprepped sigmoidoscopy vs. biopsies of the colonic mucosa at the time of an unprepped sigmoidoscopy, using alpha- and beta-diversity metrics following bacterial 16S rDNA sequencing. The findings may have relevance to health and disease states in which bacterial metabolism has a significant impact on molecules/metabolites that are recirculated between the gut lumen and mucosa and systemic circulation, such as estrogens (as in BC) or bile acids.

## 2. Methods

### 2.1. Study subjects

The subjects were recruited from Rush University and through referrals from neighboring John H. Stroger, Jr. Hospital of Cook County. Rush University Institutional Review Board (IRB; No: 08022802) approved this study and all subjects provided verbal and written informed consent prior to enrollment. Subjects were females, ages 41–74. From each subject, three different sample types were collected: at-home-collected stool (*n* = 48 total), stool collected at the time of an unprepped flexible sigmoidoscopy, i.e., endoscopically-collected stool (*n* = 48 total), and colonic biopsy samples (*n* = 48 total). Subjects filled out a structured survey in order to gather demographics and medical history prior to beginning the study. Inclusion criteria for subjects included women who have stage 0, 1, and II cancers, diagnosed within the last 6 months, with no presence of metastatic disease. Inclusion criteria for controls included race/ethnicity matched healthy females who have completed a recent mammography within the last 6 to 9 months and who have no abnormalities on their mammogram. Equal numbers were recruited from the study (*n* = 24 for BC and *n* = 24 controls that are matched to age and race). All subjects were asked to have stable dietary habits and were on a typical Western-type diet common in the United States. Subjects were excluded from the study if: (1) Age < 40 or > 80 years; (2) Any acute illness requiring immediate hospitalization; (b) Presence of symptomatically active GI disease such as inflammatory bowel disease (except hemorrhoids and hiatal hernia); (3) Pre-existent organ failure or co-morbidities as these may change GI flora (A. Liver disease (cirrhosis or persistently abnormal AST or ALT that are 2× normal), B. Kidney disease (creatinine > 2.0 mg/dL), C. Uncontrolled psychiatric illness, D. Clinically active lung disease or decompensated heart failure, E. Known HIV infection, F. Alcoholism, G. Transplant recipients, H. Diabetes); (4) Presence of short bowel syndrome or severe malnutrition with ideal body weight < 90% or obesity with BMI > 40; (5) Daily use of anticoagulation medications; (6) Antibiotic, probiotic, or prebiotic use within 1 month of enrollment; (7) Use of HRT (hormone replacement therapy); (8) Use of immunosuppressive medications within 3 months of enrollment or NSAIDs within 3 weeks of enrollment; (9) Any endoscopic, histological or past evidence of colonic or diarrheal or major gastrointestinal diseases (e.g., IBD, colitis or enteritis, colon cancer, diverticulitis).

### 2.2. Sample collection

All subjects had an unsedated, unprepped flexible sigmoidoscopy using CO2 insufflation. Subjects did not receive any bowel purgatives prior to procedure. A standard upper endoscope was used for patient comfort. No bowel cleansing was done prior to the procedure. During the sigmoidoscopy, mucosal biopsies were obtained in the distal sigmoid at about 20–25 cm from the anal verge with a sterile disposable standard 2.2 mm pinch-biopsy forceps. Care was taken to make sure that the area biopsied did not have any overlying stool. Additionally, stool specimens were obtained from the lumen of the colon during the procedure with a sterile disposable Roth net. Both samples were snap-frozen in liquid nitrogen in the endoscopy room and stored in a −80°C freezer until analysis. We also asked the subjects to provide us with a stool sample that they bring from home (within 24 h of defecation) to the procedure visit. Subjects were asked to place a disposable hat onto the toilet bowl at home before their anticipated defecation. This hat is an empty plastic container that can be placed onto the toilet bowl under the toilet seat and allows for collection of the sample without contamination from the toilet bowl. It is a standard piece of disposable plastic that is typically used for stool specimen collection clinically. The subject was asked to sit and defecate normally as they would on their home toilet bowl. The subject was asked to their stool (using several sterile tongue depressors or by inverting the hat) into a gas tight zip lock bag from BD Gas Pak EZ Pouch system. The subject was then asked to place a packet into each of the gas tight zip lock bags that absorbs oxygen, called the BD GasPak EZ Anaerobe Pouch System with Indicator. The subject was asked store stool in their home freezer until drop off of their stool specimen within 24 h to the clinical or research offices at Rush University Medical Center. During transport the subject was given a cooler bag with insulation to prevent any temperature changes. Typically subjects defecated the night before or the morning of the endoscopic procedure and brought the stool samples to their endoscopy appointment. The home-stool samples were stored in a −80°C freezer until analysis. Samples that were brought that were not frozen were not used for analysis.

### 2.3. 16S rRNA sequencing

FastDNA Spin Kit for Soil (MP Biomedicals, Solon, OH, United States) was used to extract DNA from the samples, using the manufacturer’s recommended protocol. Approximately 100 mg of stool was used for extractions. The amount of extracted DNA template from each fecal sample was verified with fluorometric quantitation (Qubit, Life Technologies, Grand Island, NY, United States). All DNA extractions resulted in >10 ng/μL DNA concentration. 16S rRNA sequencing was completed at Argonne National Labs using the V4 region primers with standard MiSeq paired-end sequencing protocols to generate 2 × 150 bp length per read. Forward primer 28F: 5′ GAGTTTGATCNTGGCTCAG 3′ and reverse primer 519R: 5′ GTNTTACNGCGGCKGCTG 3′ were used for sequencing the 16S rDNA. Input DNA for PCR protocol was 2.5 μL microbial DNA (5 ng/μL) in total volume of 25 μL. PCR process was initial denaturation at 95°C for 3 min, 25 cycles consisting of 30 s denaturation 95°C, 30 s annealing 55°C, and 30 s extension 72°C, and a final extension at 72°C for 5 min.

### 2.4. Data processing

Python scripts in Quantitative Insights Into Microbial Ecology (QIIME 2; Bolyen et al., 2019) software pipeline was used to process sequence files as ASVs (amplicon sequence variants). Sequences were filtered for low-quality samples (defined as samples with feature count < 5,000), pair-end sequences were merged, denoised, and chimera-filtered (consensus method with min abundance of potential parents of chimeric sequences were 2) using Dada2 with the following parameters: 3′ forward and reverse sequences were truncated at 149 bps; and 5′ forward and reverse sequences were trimmed at 13 bps. There were 48 subjects remaining after low-quality samples were removed. Rare and low-abundance taxa were filtered out (ASVs with min frequency < 10). Taxonomy assignment was performed against the Greengenes database (v.13.8.99; DeSantis et al., 2006) using QIIME2’s built-in (Naive Bayes) classifier (Bokulich et al., 2018). Further filtering removed ASVs if they classified as “Mitochondria,” “Chloroplast,” “Burkholderiales,” “Rickettsiales,” or “Archaea” (except *Methanobrevibacter* and *Methanosphaera*) or were unclassified at Kingdom or Phylum level.

### 2.5. Statistics

Alpha diversity and its statistical analysis were calculated using QIIME2 and ASV level. For alpha diversity analysis, samples were rarified to a sampling depth of 5,000. For beta diversity, multivariate reduction analyses using principal coordinates with a weighted Unifrac metric, unweighted Unifrac metric and Bray-Curtis metric in QIIME2 were calculated to determine the global microbiome composition. Permutational multivariate analysis of variance (PERMANOVA) implementation in QIIME2 was used to perform a randomization test of significance of pseudo *F* values, with 999 randomizations for each model, on rarified sequence data and was used to statistically assess differences in beta diversity. Relative abundances were normalized using total sum scaling (TSS). Microbiome Multivariable Association with Linear Models (MaAsLin2), which adjusts for multiple comparisons and determines multivariable associations between clinical metadata and microbial features, was used to compare taxa abundance between groups (Mallick et al., 2021). PICRUSt2 was used to determine functional pathways (Douglas et al., 2020). Graphs of the data were generated in R. SPSS (V24.0.0, Chicago, IL, United States) was used to analyze clinical metadata. QIIME2 was used to analyze alpha and beta diversity data, using pairwise Kruskal-Wallis. In SPSS, Kruskal-Wallis was used for the other comparisons. Scikit-bio V0.5.7, Microsoft Excel and PowerPoint, MatPlotLib (Hunter, 2007), GraphPad Prism V9.4.1 for Windows and MacOS (La Jolla, California, United States) were also used to generate plots.

## 3. Results

### 3.1. Subject and sample characteristics

All subjects included in the study were female (*n* = 48). Of the subjects, 24 had breast cancer and 24 were healthy controls. The ages of subjects ranged from 41 to 74 years, with the mean being 56 years of age. The racial and ethnic distribution of the subjects are given in Table 1. Subjects provided each of the three sample types (*n* = 48 for at-home-stool, 48 for colonic biopsy, 48 for endoscopically-collected-stool). Sample types were then compared to each other, with the subjects being their own control.

**Table 1:** Subject characteristics.

	All subjects
	*n* = 48
Mean age in years (stdev)	56.3 (7.2)
Mean BMI (stdev)	28.9 (5.3)
**Period status**	
Post-menopausal [*n*(%)]	34 (64.2)
Peri-menopausal [*n*(%)]	6 (12.5)
Pre-menopausal [*n*(%)]	8 (16.7)
**Race**	
Asian [*n*(%)]	4 (8.3)
Black or African American [*n*(%)]	19 (39.6)
White [*n*(%)]	25 (47.2)
**Ethnicity**	
Hispanic/Latino [*n*(%)]	1 (2.1)
Non-Hispanic/Latino [*n*(%)]	47 (97.9)

### 3.2. Sample type was associated with differences in alpha diversity

Illumina sequencing of the V4 hypervariable region of 16S rRNA amplicons from all individual samples yielded 9,902,211 raw reads and 6,285,387 reads after pre-processing. The range of the sequence counts per sample was from 5,490 to 435,122 reads. In total, 2,307 ASVs were detected. We first analyzed alpha-diversity in bacterial communities using the Chao1, Pielou’s Evenness, Faith Phylogenetic diversity (Faith PD), Shannon index and Simpson. Overall, all sample types significantly differ by all of the alpha-diversity metrics when compared in a three-way analysis, suggesting differences between communities within each sample type (*p* < 0.001 for all metrics).

**Figure 1:**
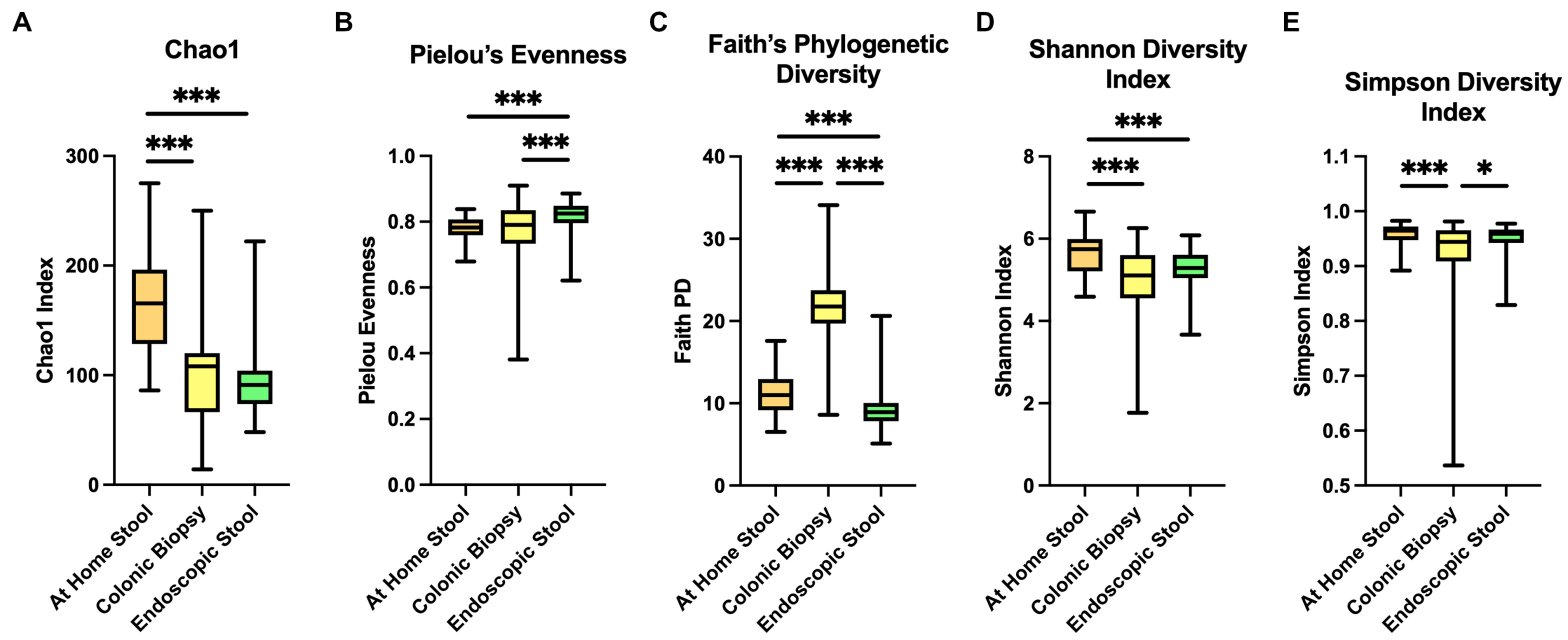
Alpha diversity indices between sample types. At-home stool samples are in orange (left), colonic biopsy samples are in yellow (middle), and endoscopically-collected stool samples are in green (right). X-axis shows sample type. Y-axis shows the value of each diversity index. **(A)** Chao 1 index, **(B)** Pielou’s Evenness, **(C)** Faith’s Phylogenetic Diversity, **(D)** Shannon diversity index, and **(E)** Simpson diversity index. *p < 0.05, **p < 0.01, **p < 0.001.

The Chao1 showed higher alpha-diversity in the at-home stool group compared to both colonic biopsy (*p* < 0.001) and endoscopically-collected stool samples (*p* < 0.001; Figure 1A). Pielou’s evenness was also significantly higher in endoscopically-collected stool samples compared to both at-home stool samples and colonic biopsy samples (Figure 1B; *p <* 0.001 and *p* = 0.009, respectively). Biopsy samples had the largest range in evenness overall (Figure 1B) and did not differ in evenness from at-home collected stools (*p* = 0.442). Using Faith PD, sample types were significantly different from one another in all two-way comparisons (Figure 1C; *p* < 0.001); highest diversity was seen in the colonic biopsies, followed by at-home stools. The lowest diversity was seen in the endoscopically-collected stools.

In Shannon index, both colonic biopsy and endoscopically-collected stool samples showed significantly lower alpha-diversity compared to at-home-collected stool samples (Figure 1D; *p* < 0.001 and *p* < 0.001 respectively). Simpson’s index showed significantly lower diversity in colonic biopsy samples compared to at-home stool samples (*p* < 0.001), and modestly lower diversity compared to endoscopically-collected stool samples (Figure 1E; *p* = 0.022).

### 3.3. Overall differences in microbiota composition were seen between sample type groups

**Figure 2:**
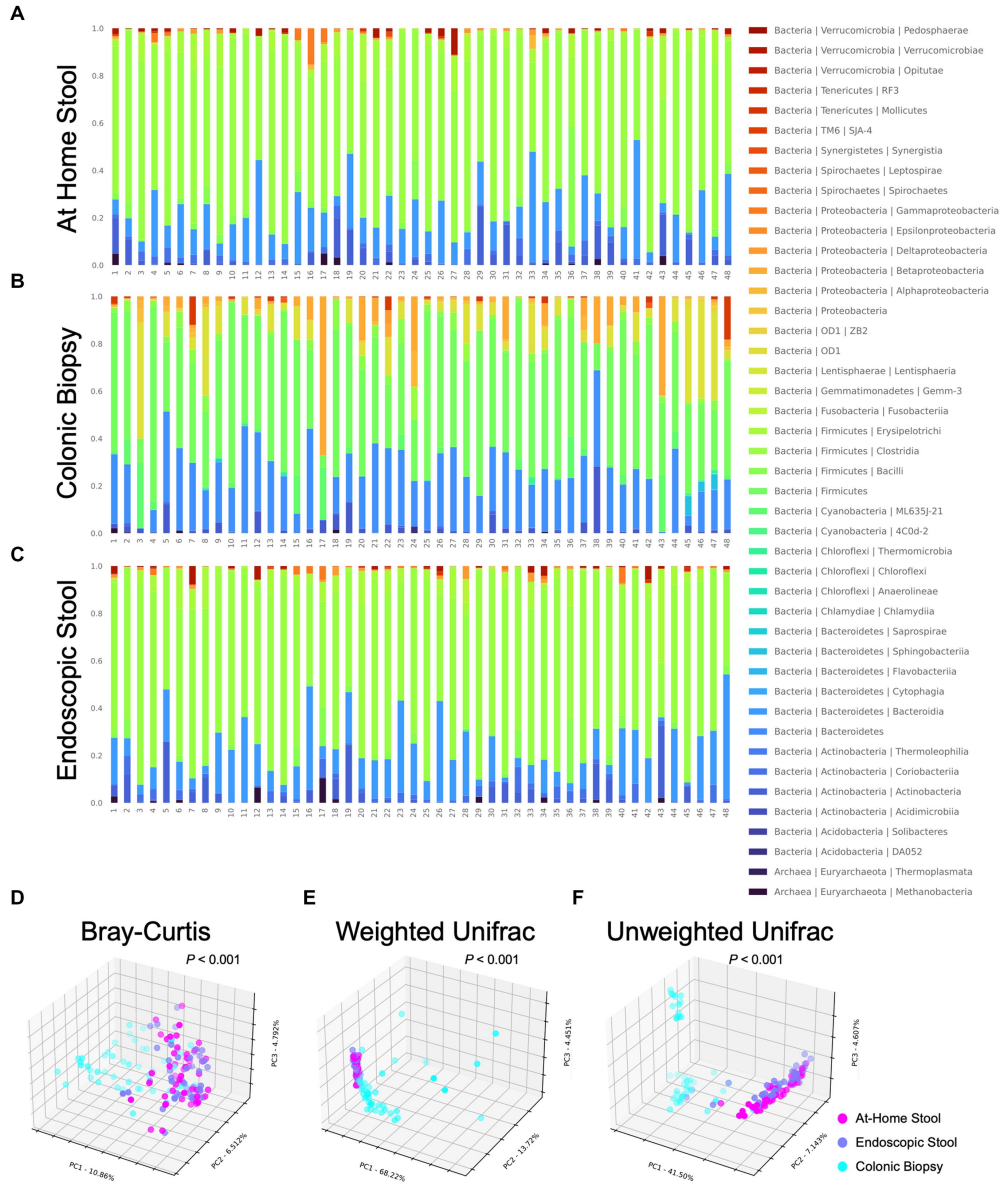
Taxonomic barplots and beta diversity indices between sample types using 3D principal coordinates analysis (PCoA) with Bray-Curtis metric, weighted Unifrac and unweighted Unifrac. Each bacterial class is represented in a different color. At-home collected stool and endoscopically-collected stool show distinct visual similarities compared to colonic biopsy samples. **(A)** At-home stool, **(B)** colonic biopsies, and **(C)** endoscopic stool. Each dot represents a single subject’s sample. At-home samples are shown in pink, biopsy samples are shown in blue, and endoscopic samples are shown in purple. Visual separation between stool samples (at-home and endoscopic) and biopsy samples is apparent. **(D)** Bray-Curtis (*p* < 0.001), **(E)** Weighted Unifrac (*p* < 0.001), and **(F)** Unweighted Unifrac (*p* < 0.001).

Differences in microbiome composition between sample types are seen on barplots representing relative abundance of taxa at the phylum and class levels (Supplementary Figure 1; Figure 2). At-home stool and endoscopic stool look more visually similar in composition compared to biopsy samples (Figures 2A–C wherein there is a clear change in bacterial taxa denoted in red to yellow color groups).

In order to quantify the overall differences in bacterial composition between sample types, beta-diversity between each sample was calculated using the Bray-Curtis metric, weighted and unweighted UniFrac. Unifrac metrics entail assigning samples on a phylogenetic tree and measuring distances between sets of taxa within a sample based on the fraction of the branch length of the tree that divides into descendants. Taxa that are phylogenetically related cause lower (i.e., less divergent) UniFrac values, while distant taxa on varying branches of the phylogenetic tree cause larger differences. Samples were then ordinated (i.e., grouped) with principal coordinates analysis (Figure 2). In the graphs, each sample is seen as a single dot, and samples that are more similar in overall bacterial composition appear closer or cluster together.

When the three sample types were different in overall composition for all of the metrics examined (*p* < 0.001 all using PERMANOVA). Biopsy samples had a significantly different bacterial composition by all three metrics when compared to at-home or endoscopically-collected stool samples (*p* < 0.001, < 0.001, < 0.001, respectively, for Bray-Curtis, weighted Unifrac and unweighted Unifrac using PERMANOVA). Specifically, when looking at the 3D distribution of biopsy samples, more cases can be seen skewing to the right (Figures 2D,F) or left (Figure 2E) of the graph indicating differences in both rare and abundant taxa as well as phylogenetically-diverse taxa in the biopsy samples compared to both types of stool samples (i.e., luminal samples). On the other hand, endoscopically-collected and at-home stool samples can be seen clustering more closely together suggesting that they are visually more similar to each other using all three metrics (Figures 2D–F).

Further statistical analysis of these visual changes observed using PERMANOVA showed no differences between the at-home vs. endoscopically-collected stool samples using the Bray-Curtis (*p* = 0.993) or weighted UniFrac (*p* = 0.974) metrics (Figures 2A,B). Whereas looking at the unweighted Unifrac analysis (Figure 2C), at-home and endoscopically-collected stool can be seen distinctly clustered further apart. Beta-diversity was also statistically significantly different in at-home vs. endoscopically-collected stool samples with the unweighted UniFrac metric (*p* < 0.001). Considering a higher influence of rare taxa on the unweighted Unifrac (which primarily conducts a presence and absence analysis), these differences suggest the presence of phylogenetically-diverse rarer organisms account for the differences between endoscopically-collected stool samples vs. that of home-collected ones. Using unweighted Unifrac, endoscopically-collected stool samples resemble neither the biopsy samples nor at-home stool samples. For rare organisms, the stool types are thus not exactly comparable. This clustering can also clearly be seen in 2D PCoA plots of beta diversity analysis (Supplementary Figure 2).

The variation was further assessed by examining interpersonal Weighted Unifrac distances within each sample type. Unifrac distances within colonic biopsy samples were significantly higher than those within at-home stool or endoscopically-collected stool samples (1.172 ± 0.850 vs. 0.247 ± 0.062 vs. 0.259 ± 0.080 respectively; *p* < 0.0001 and *p* < 0.0001 respectively). However, there was not a significant difference between interpersonal Unifrac distances when comparing at-home collected stool to endoscopically-collected stool (*p* = 0.997).

### 3.4. Different sample types were associated with specific bacteria genera

**Figure 3:**
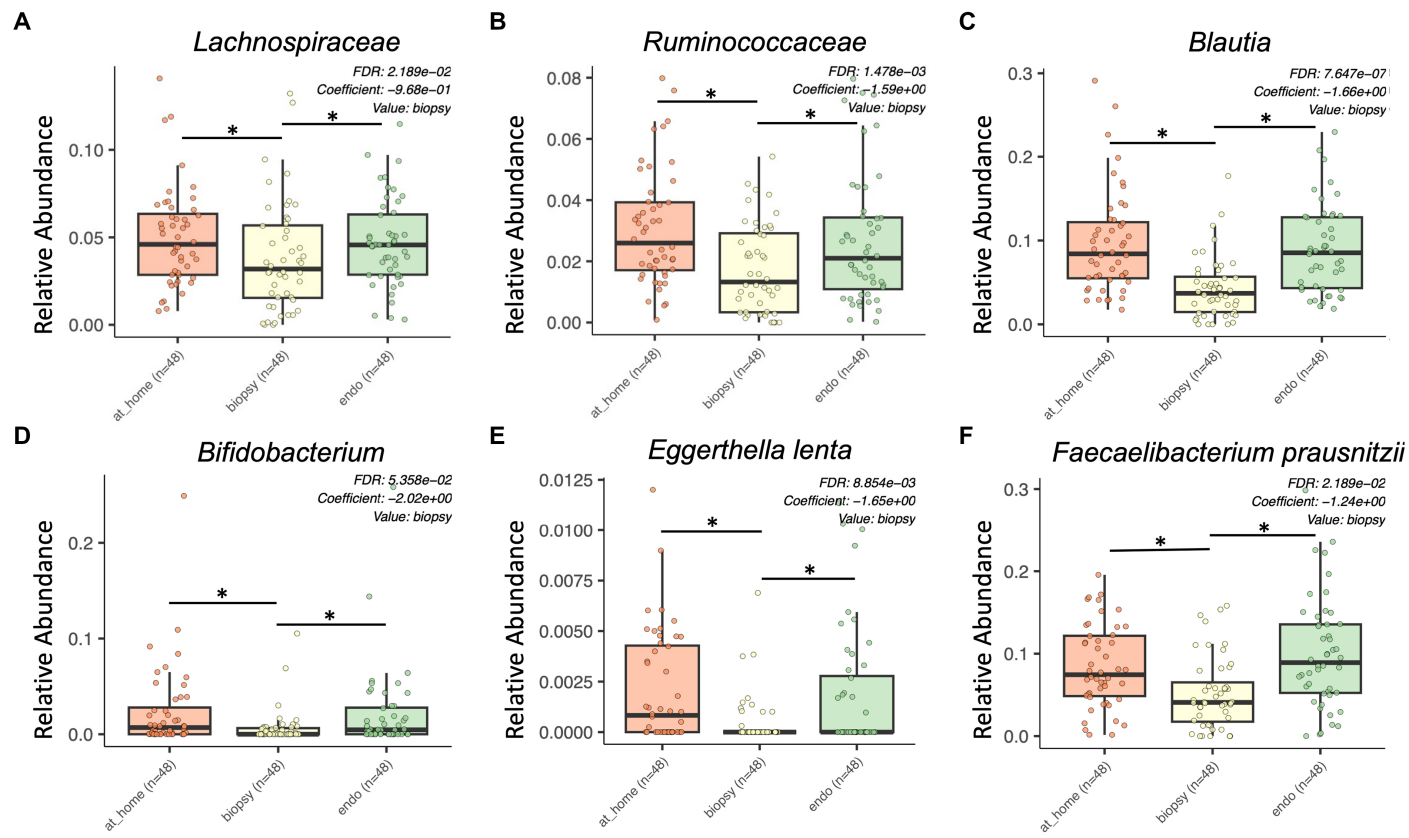
Bacterial taxa enriched in stool sample types by MaAsLin2 analysis. At-home stool samples are in orange (left), colonic biopsy samples are in yellow (middle), and endoscopically-collected stool samples are in green (right). X-axis shows sample type. Y-axis shows relative abundance for each described taxon. Only statistically significant enriched taxa are shown for all panels (*p* < 0.001, q-value < 0.05). **(A)** *Lachnospiraceae*, **(B)** *Ruminococcaceae*, **(C)** *Blautia*, **(D)** *Bifidobacterium*, **(E)** *Eggerthella lenta*, and **(F)** *Faecalibacterium prausnitzii*.

All three sample types were compared in a three-way analysis using MaAsLin2 (*n* = 48). Here, there were 61 differentially enriched taxa between sample types (defined as *p* < 0.01, q-value < 0.05; Figure 3). The majority of enriched taxa were seen in stool samples (both at-home and endoscopically-collected stool). There were higher levels of Proteobacteria in biopsy samples, with significantly more Actinobacteria and Firmicutes in stool (all *p* < 0.001, q-value < 0.05).

*Lachnospiraceae* were significantly enriched in both at-home stool samples and endoscopically collected stool samples (Figure 3A). Additionally, there were multiple genera and species within the *Lachnospiraceae* family enriched in stool samples, including *Blautia, Eggerthella lenta, Coprococcus, Anaerostipes, Roseburia faecis,* and *Dorea* (Figures 3C,E; Supplementary Figures 3A,B). Further, *Ruminococcaceae*, and some genera within the family such as *Faecalibacterium prausnitzii, Ruminococcus, Butyricicoccus pullicaecorum,* and *Gemmiger formicilis,* were all significantly enriched in stool samples types compared to colonic biopsies (Figures 3B,F; Supplementary Figures 3C–E). Finally, *Bifidobacterium* and *Clostridium spiroforme* were also significantly enriched in stool samples (Figure 3D).

There were also some taxa specifically enriched in at-home stool samples, highlighting some of the differences in specific taxa between stool sample types. *Actinomyces, Aldercreutzia,* and *Holdemania* were all significantly enriched specifically in at-home-collected stool samples (Supplementary Figures 3F–H). Alternatively, there were no bacterial taxa significantly enriched specifically in endoscopically-collected stool samples.

**Figure 4:**
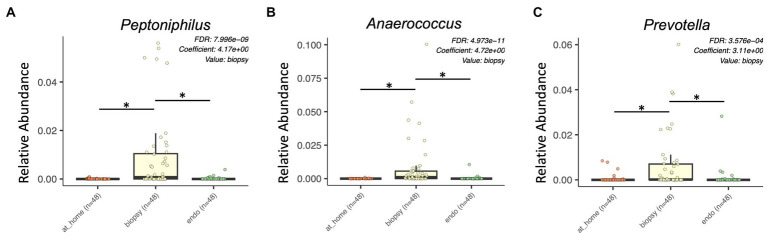
Bacterial taxa enriched in colonic biopsy samples by MaAsLin2 analysis. At-home stool samples are in orange (left), colonic biopsy samples are in yellow (middle), and endoscopically-collected stool samples are in green (right). X-axis shows sample type. Y-axis shows relative abundance for each described taxon. Only statistically significant enriched taxa are shown for all panels (*p* < 0.001, q-value < 0.05). **(A)** *Peptoniphilus*, **(B)** *Anaerococcus*, and **(C)** *Prevotella.*

Finally, there were multiple specific bacterial taxa significantly enriched in colonic biopsies. In the *Tissierellaceae* family, *Anaerococcus, Finegoldia, Peptoniphilus, and Gallicola* were all significantly enriched in colonic biopsy samples (Figures 4A,B; Supplementary Figure 3I). Additional taxa significantly enriched in colonic biopsies included *Prevotella, Porphyromonas, Corynebacterium,* and *Campylobacter* (Figure 4C).

Overall, there were significant differences in relative abundance between sample types, showing higher relative abundance of *Lachnospiraceae* and *Ruminococcaceae* in stool and higher abundances of *Tisserellaceae* in biopsy samples.

### 3.5. PICRUSt2 showed differences in functional pathways between sample types

**Figure 5:**
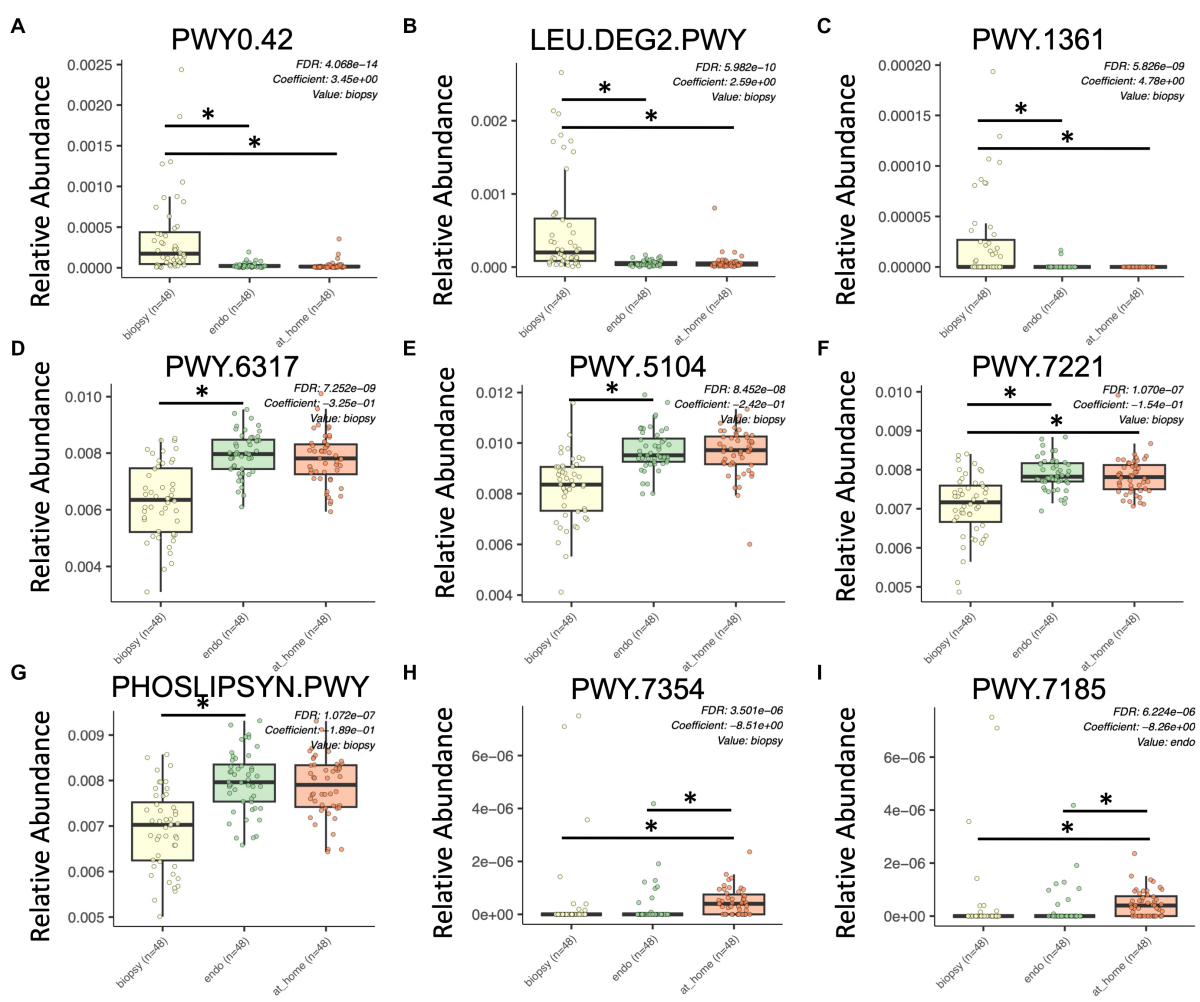
Significant differences in functional pathways between sample types using PICRUSt2. Colonic biopsy samples are in yellow (left), endoscopically-collected stool samples are in green (middle), and at-home stool samples are in orange (right). X-axis shows sample type. Y-axis shows relative abundance for each described functional pathway. Only statistically significant enriched functional pathways are shown for all panels (*p* < 0.0001, q-value < 0.15). **(A)** PWY0.42, 2-methylcitrate; **(B)** LEU.DEG2.PWY, L-leucine degradation; **(C)** PWY.1361, benzoyl-CoA degradation; **(D)** PWY.6317, D-galactose degradation; **(E)** PWY.5104, L-isoleucine biosynthesis; **(F)** PWY.7221, guanosine ribonucleotide *de novo* biosynthesis; **(G)** PHOSLIPSYN.PWY, phospholipid biosynthesis; **(H)** PWY.7354, alacinomycine biosynthesis; and **(I)** PWY.7185, UTP and CTP dephosphorylation.

PICRUSt2 (Phylogenetic Investigation of Communities by Reconstruction of Unobserved States) in QIIME2 was used to estimate the functional profile of the microbiome. PICRUSt2 uses 16S rRNA sequences to analyze the metagenome of bacteria in samples by estimating the abundance of gene families in order to determine composition. Here, we used MaAslin2 to determine differences in functional pathways between at-home collected stool, colonic biopsies, and endoscopically collected stool in a pairwise analysis. This analysis discovered 258 significantly different pathways across sample types (defined as *p* < 0.015, q-value < 0.05; Figure 5).

Of the most significantly enriched in colonic biopsy samples were PWY0.42 (2-Methylcitrate cycle), LEU.DEG2.PWY (L-leucine degradation), and PWY.1361 (benzoyl-CoA degradation I; Figures 5A–C). Alternatively, PWY.6317 (D-galactose degradation), PWY.5104 (L-isoleucine biosynthesis IV), PWY.7221 (guanosine ribonucleotide *de novo* biosynthesis), and the phospholipid biosynthesis pathway were all significantly enriched in stool samples (both in at-home and endoscopically-collected; Figures 5D–G). PWY.7354 (alacinomycin biosynthesis) and PWY.7185 (UTP and CTP dephosphorylation I) were specifically enriched in at-home collected stool samples (Figures 5H,I). In terms of functional pathways, stool samples appear more similar in function than endoscopically collected samples. This highlights the differences between endoscopically collected stool and at-home collected stool.

### 3.6. Contribution of clinical factors to microbiome composition between sample types

Adonis was used to determine the contribution of clinical factors, including BMI, age, race, and period status to the composition of the microbiome between sample types. When looking at the overall dataset, sample type contributed significantly to the microbiome composition compared to other clinical factors. The R^2^ of sample type was 0.359 and 0.360 for the weighted and unweighted Unifrac metrics respectively, with a Pr(>F) of 0.001 for both metrics. This R^2^ value is much higher than that of the studied clinical variables, which all had R^2^ values of less than 0.02 (Table 2). When further split by sample type, these clinical factors continued to have a negligible contribution to microbiome composition, all with R^2^ values below 0.06. The R^2^ for Bray-Curtis metric was 0.071 with a Pr(>F) of 0.001.

Contribution of clinical factors to the microbiome was further assessed by examining beta diversity statistics between clinical variable groups, in addition to looking at beta diversity correlations with continuous variables such as BMI and age (Table 2; Supplementary Tables 1, 2). When adjusted for multiple comparisons, none of these differences were significant (all *p* > 0.05). In unadjusted comparisons, there were minor differences noted, although the differences were not consistent across multiple metrics: in colonic biopsies, black and white subjects had significantly different microbiome composition when looking at the Bray-Curtis metric only (*p* = 0.037; Supplementary Table 1). However, this difference was not seen in either stool sample type or any of the Unifrac metrics. Additionally, endoscopically-collected stool was significantly correlated with BMI only in the Weighted Unifrac (*p* = 0.043) and age in the Unweighted Unifrac (*p* = 0.032; Supplementary Table 2) when testing with Spearman’s rank correlation. These correlations were not seen in either at-home collected stool or colonic biopsies. Thus, the clinical variables did not consistently explain a significant portion of the variability in any of the sample types across three studied beta diversity metrics.

**Table 2:** Clinical variable adonis statistics by sample type.

	At-home stool		Colonic biopsy		Endoscopic stool	
	R^2^	Pr(>F)	R^2^	Pr(>F)	R^2^	Pr(>F)
**Bray-Curtis**						
BMI	0.027	0.124	0.021	0.416	0.017	0.825
Age	0.029	0.083	0.019	0.578	0.026	0.138
Race	0.041	0.566	0.053	0.092	0.046	0.267
Period status	0.034	0.931	0.046	0.298	0.044	0.380
**Weighted Unifrac**						
BMI	0.028	0.194	**0.036**	**0.255**	0.031	0.098
Age	**0.043**	**0.045**	0.003	0.844	0.032	0.093
Race	0.037	0.554	0.026	0.611	0.035	0.537
Period status	0.032	0.647	0.029	0.577	0.042	0.327
**Unweighted Unifrac**						
BMI	0.031	0.061	**0.049**	**0.029**	0.025	0.185
Age	0.025	0.202	0.011	0.974	0.027	0.106
Race	0.042	0.387	0.049	0.316	0.051	0.093
Period status	0.036	0.680	0.035	0.776	0.042	0.309

## 4. Discussion

Herein we analyzed three different methods of sampling the gut microbiome, looking at at-home collected stool, colonic biopsy, and endoscopically-collected stool samples in the largest sample set reported to date. Both at-home and endoscopically-collected stool samples (i.e., luminal samples) significantly differed when compared to biopsy samples (i.e., mucosa-associated samples) in nearly all metrics examined. These findings are congruent with the current literature that fecal and mucosal microbiomes have different microbial communities (Hong et al., 2011; Stearns et al., 2011; Ringel et al., 2015; Flynn et al., 2018; Vaga et al., 2020). The differences in luminal and mucosal microbiota niches may be due to the different environments. For example, higher oxygen tension in the mucosal environment may account for the increased relative abundance of aerotolerant bacteria from the Proteobacteria phylum (Albenberg et al., 2014). Further, differences in nutrient availability, natural antibacterial components (i.e., defensins and secretory IgA), and intestinal mucosal factors (i.e., mucins) and immune cells within the mucosa may also affect the microbial community (Meade and O’Farrelly, 2018; Rowland et al., 2018; Paone and Cani, 2020). Thus, our findings suggest that both luminal and biopsy samples are helpful to get a complete picture of the gut colonic microbiome in health and disease. However, it is important to note the invasive nature of collecting biopsy samples from patients. Examining both mucosa-associated and luminal samples may be especially important if the mucosa-associated microbiome is expected to have a significant impact on the disease pathogenesis or relevant metabolites generated and/or absorbed.

In biopsy samples, there was significantly higher variation in Weighted Unifrac distances from one subject to another. Furthermore, this variation was present in both rare and abundant taxa (as evident from highly dispersed cases in all beta diversity metric graphs). Taxa that were more abundant in colonic biopsy samples were sometimes several-fold higher than their relative abundances in luminal samples, suggesting that these taxa may be of greater importance at the mucosal level in both health and disease. Within the biopsy samples, *Tissierellaceae* (i.e., *Anaerococcus* and *Peptoniphilus*) and *Prevotella* seem to be most affected and have highly differing interpersonal abundance (Figure 3). Studies or hypotheses looking at members of *Tissierellaceae* or *Prevotella* (as potential culprits of disease or preservation of health) could thus be more likely to note changes in colonic biopsy samples.

In luminal sample types, our study also notes a considerable difference in the Chao1 index, Faith’s PD and unweighted Unifrac metrics in at-home stool samples compared to endoscopically-collected samples: rarely-abundant and/or phylogenetically-diverse bacterial taxa are present in at-home collected stools. These differences between at-home stool samples and endoscopically-collected stool samples have not been reported before. To date, there is only one published study that has compared at-home-collected to endoscopically-collected stool samples (Flynn et al., 2018). But the latter study reported differences in biodiversity using a weighted, count-based metric (theta YC differences), which is unlikely to be affected by rare taxa or phylogenetically diverse taxa. In general, while the bulk of the bacterial community is expected to create the bulk of the functionality of the gut microbiome, it is well known that rare bacterial taxa can have unique functions, and presence/absence of such rare bacterial taxa can directly affect results of studies examining them: examples of such rare taxa (which constitute less than 1% of the total microbiota) with unique functions include *C. scindens*, that can synthesize secondary bile acids (that can be toxic or protective, depending; Yoshimoto et al., 2013; Marion et al., 2018; Kang et al., 2019) and can convert glucocorticoids to androgens (Ridlon et al., 2013); and adherent-invasive *E. coli* strains that plays a role in inflammation in the bowel (Kamali Dolatabadi et al., 2021; López-Siles et al., 2022). It is plausible that the higher biodiversity in the at-home stool samples in our study occurs either as a result of contamination with skin bacteria during excretion through the anal canal and/or environmental organisms during collection; and/or as a result of growth selection for some bacterial ASVs that are tolerant to lower temperatures and higher oxygen levels outside of the body. Lack of some of the rare taxa in endoscopically-collected stool samples compared to at-home ones also suggests that endoscopy equipment is unlikely to be a significant source of variation in microbiota studies. Regardless, endoscopically-collected stool was not different than at-home collected stool for abundant bacteria reflected in the Weighted Unifrac and Bray-Curtis metrics in our study. This finding is similar to that of Flynn et al. which also suggests abundant taxa are similar between the two types of stool samples. Thus, studies or hypotheses looking at abundant taxa could focus on either of these two luminal sample types.

Some of the differences in specific bacterial taxa between sample types could be important specifically to research focusing on the gut microbiome and breast cancer. For example, *Lachnospiraceae* and *Ruminococcaceae*, which were both enriched in stool samples, have been shown to be significantly enriched in the stool samples of breast cancer patients in previous studies (Goedert et al., 2015, 2018; Terrisse et al., 2021). *Aldercreutzia,* a bacterial taxon known to produce equol (which is a phytoestrogen commonly derived from soy in the diet) was more abundant in at-home collected fecal samples, compared to endoscopically collected samples. However, no studies have been done examining the microbiome of colonic biopsies in BC patients, and any specific bacterial species are reported to be enriched in BC have only been shown in stool samples. Alternatively, *Prevotella*, which has instead been shown to be enriched in breast tissue of BC patients (Urbaniak et al., 2016), was found to be significantly enriched in colonic biopsy samples compared to both at-home and endoscopically collected stool. Thus, to study hypotheses related to *Prevotella* in the gut and breast microbiome of BC patients, biopsy samples may be more likely to exhibit major changes. Outside of studies specifically investigating the microbiome in BC patients, multiple bacterial species have been shown to be involved in estrogen metabolism in the gut through the action of bacterial glucuronidase enzymes. The beta-glucuronidase gene (GUS) is present in a wide variety of bacterial species, including being found in more than half of Firmicutes (Dabek et al., 2008). Some species well known for their glucuronidase activity are *Ruminococcus* and *Bifidiobacterium*, both of which were found to be significantly enriched in stool samples in our study. Additionally, some specific species of *Clostridium, Lactobacillus,* and *Bacteroides* are known to have GUS activity (Beaud et al., 2005; Pollet et al., 2017). GUS enzymes can have substrate specificity and it is currently unclear which of these taxa’s GUS enzymes would be most relevant to estrogen deconjugation within the gut, and whether the sum of all of them are collectively important. GUS specific taxa were not noted to be enriched in biopsy samples.

There are several strengths of the current study: this study looked at a large number of subjects (*n* = 48) compared to many of the studies that have been previously published (Zoetendal et al., 2002; Carroll et al., 2010; Momozawa et al., 2011; Ringel et al., 2015; Shobar et al., 2016; Altomare et al., 2019; Kim et al., 2021; Jiao et al., 2022). The study also examined samples that have not been subjected to bowel cleansing. Most studies analyzing the mucosa-associated microbiome performed biopsy sampling after bowel cleansing. Bowel cleansing preparations prior to the sampling procedure have been shown to have several negative effects on the intestinal microbial community, reducing the microbiome diversity (Shobar et al., 2016; Nagata et al., 2019; Powles et al., 2022). This is because bowel cleansing prior to the colonoscopy procedure causes sampled luminal content to be largely derived from mucus-associated microbes instead of the bulk stool stream. At the class level, bowel cleansing prior to the sampling procedure can lead to increases in Proteobacteria and Coriobacteria, with a significant decrease in Clostridia (Jalanka et al., 2015). Additionally, by not washing out bacteria prior to sampling, detection of low-abundance taxonomic groups within luminal contents and colonic mucosal biopsies is more likely (Powles et al., 2022). While there are few reports on the specific effects of bowel cleansing on the microbiome, there is one published study that examined both colonic biopsies and luminal samples before and after bowel preparation (Shobar et al., 2016). Here, the mucosal and luminal samples were significantly different prior to cleansing, but bacterial OTUs became more comparable in mucosal and luminal samples after cleansing.

Our study sample was limited to middle-aged and older women at screening age for BC. It is thus unknown whether the findings are applicable to all subjects. While the gut microbiome is thought to play an important role in the enterohepatic circulation of estrogens, and therefore potentially impact BC (Plottel and Blaser, 2011; Kwa et al., 2016), no studies have examined what types of samples would be best suited for these studies in women. This is the only study to date that examines the colonic mucosal microbiome in women at screening age for BC and provides important insights into the differences between sampling methods for analyzing the gut microbiome. So far, all studies assessing the gut microbiome in women at risk for BC have only taken into consideration fecal samples, commonly collected by the subjects at home (Aarnoutse et al., 2021; Bobin-Dubigeon et al., 2021; Byrd et al., 2021). Differences have been noted in both rare and common bacterial taxa.

In conclusion, microbiota associated with biopsy samples should be separately analyzed from that found within at-home and endoscopically-collected stool samples. While at-home and endoscopic stool samples are more similar and could be combined in analysis for hypotheses examining abundant bacteria, it is important to be aware of potential differences in rare and phylogenetically distinct bacterial taxa between at-home collected and endoscopically-collected stool samples. Enriched abundance of some rare bacterial taxa in at-home collected stool samples could be advantageous in the study of some of these rare taxa but could also be a source of enhanced phylogenetic variation in bacterial composition which may detract from reaching definitive conclusions about such taxa. In general, stool samples appear to adequately represent bacterial taxa with GUS activity.

## Data availability statement

The original contributions presented in the study are publicly available. This data can be found here: https://www.ncbi.nlm.nih.gov/bioproject/PRJNA936897.

## Ethics statement

The studies involving human participants were reviewed and approved by Rush University Medical Center IRB. The patients/participants provided their written informed consent to participate in this study.

## Author contributions

EM conceived and designed the experiments, collected the samples, edited, and reviewed the manuscript. PE performed the experiments. CN analyzed all experimental data, wrote, and edited the manuscript. LR edited and reviewed the manuscript. AM, TW, TL, and MC contributed to patient recruitment and clinical data collection. All authors contributed to the article and approved the submitted version.

## Funding

This work was supported by the NIH/NCATS R21TR003105 grant and CDMRP BCRP BC074932 grant to EM. LR was supported through TL1 5TL1TR002388-04.

## Conflict of interest

The authors declare that the research was conducted in the absence of any commercial or financial relationships that could be construed as a potential conflict of interest.

## Publisher’s note

All claims expressed in this article are solely those of the authors and do not necessarily represent those of their affiliated organizations, or those of the publisher, the editors and the reviewers. Any product that may be evaluated in this article, or claim that may be made by its manufacturer, is not guaranteed or endorsed by the publisher.

## Supplementary material

### SUPPLEMENTARY FIGURE S1

Taxonomic barplots at the phylum levels between sample types.Each bacterial class is represented in a different color. At-home collected stool and endoscopically-collected stool show distinct visual similarities compared to colonic biopsy samples. **(A)** At-home stool **(B)** Colonic biopsies **(C)** Endoscopic stool.

### SUPPLEMENTARY FIGURE S2

Beta diversity indices between sample types using 2D principal coordinates analysis (PCoA) with Bray-Curtis metric, weight Unifrac, and unweighted Unifrac. Each dot represents a single subject’s sample. At-home samples are shown in pink, biopsy samples are shown in blue, and endoscopic samples are shown in purple. Bray-Curtis metric is shown in the first row, weighted Unifrac is shown in the second row, unweighted Unifrac is shown in the third row. PC1 vs. PC2 can be seen in the first column, PC1 vs. PC3 is shown in the second column, and PC2 vs. PC3 is shown in the third column. **(A)** Bray-Curtis PC1 vs. PC2 **(B)** Bray-Curtis PC1 vs. PC3 **(C)** Bray-Curtis PC2 vs. PC3 **(D)** Weighted Unifrac PC1 vs. PC2 **(E)** Weighted Unifrac PC1 vs. PC3 **(F)** Weighted Unifrac PC2 vs. PC3 **(G)** Unweighted Unifrac PC1 vs. PC2 **(H)** Unweighted Unifrac PC1 vs. PC3 **(I)** Unweighted Unifrac PC2 vs. PC3.

### SUPPLEMENTARY FIGURE S3

Differentially abundant bacterial taxa compared between sample types using MaAsLin2. At-home stool samples are in orange (left), colonic biopsy samples are in yellow (middle), and endoscopically-collected stool samples are in green (right). X-axis shows sample type. Y-axis shows relative abundance for each described taxon. Only statistically significant enriched taxa are shown for all panels (*P* < 0.001, q-value < 0.05). **(A)** Coprococcus **(B)** Anaerostipes **(C)** Ruminococcus **(D)** Gemmiger formicilis **(E)** Butyricicoccus pullicaecorum **(F)** Actinomyces **(G)** Adlercreutzia **(H)** Holdemania **(I)** Finegoldia.
