# Supplementary material for: Comparison of gut microbiome composition in colonic biopsies, endoscopically-collected and at-home-collected stool samples
Source: Front Microbiol. 2023 Jun 1;14:1148097. doi: 10.3389/fmicb.2023.1148097 (PMC10264612; doi:10.3389/fmicb.2023.1148097)
Supplement: Supplementary file 1 [file Table_1.docx]

## Supplemental Table 1. Clinical Variable Beta Diversity Statistics by Sample Type.

|  | Group1 | Group2 | At-Home Stool | Colonic Biopsy | Endoscopic Stool |
| --- | --- | --- | --- | --- | --- |
| PeriodStatus |  |  |  |  |  |
| Bray-Curtis | Peri | Post | 0.416 | 0.067 | 0.137 |
|  | Peri | Pre | 0.578 | 0.438 | 0.551 |
|  | Post | Pre | 0.394 | 0.596 | 0.148 |
|  | Overall |  | 0.446 | 0.236 | 0.128 |
| Weighted Unifrac | Peri | Post | 0.801 | 0.477 | 0.446 |
|  | Peri | Pre | 0.561 | 0.220 | 0.446 |
|  | Post | Pre | 0.302 | 0.430 | 0.078 |
|  | Overall |  | 0.576 | 0.486 | 0.170 |
| Unweighted Unifrac | Peri | Post | 0.598 | 0.443 | 0.227 |
|  | Peri | Pre | 0.613 | 0.556 | 0.854 |
|  | Post | Pre | 0.140 | 0.993 | 0.326 |
|  | Overall |  | 0.330 | 0.872 | 0.316 |
|  |  |  |  |  |  |
| Race |  |  |  |  |  |
| Bray-Curtis | Black | White | 0.467 | **0.036** | 0.302 |
| Weighted Unifrac | Black | White | 0.216 | 0.223 | 0.691 |
| Unweighted Unifrac | Black | White | 0.461 | 0.100 | 0.280 |

Bolded values indicate *p* < 0.05.
