# Supplementary material for: Comparison of gut microbiome composition in colonic biopsies, endoscopically-collected and at-home-collected stool samples
Source: Front Microbiol. 2023 Jun 1;14:1148097. doi: 10.3389/fmicb.2023.1148097 (PMC10264612; doi:10.3389/fmicb.2023.1148097)
Supplement: Supplementary file 2 [file Table_2.docx]

## Table 2. Clinical Variable Adonis Statistics by Sample Type.

|  | At-Home Stool |  | Colonic Biopsy |  | Endoscopic Stool |  |
| --- | --- | --- | --- | --- | --- | --- |
|  | R^2^ | Pr(>F) | R^2^ | Pr(>F) | R^2^ | Pr(>F) |
| Bray-Curtis |  |  |  |  |  |  |
| BMI | 0.027 | 0.124 | 0.021 | 0.416 | 0.017 | 0.825 |
| Age | 0.029 | 0.083 | 0.019 | 0.578 | 0.026 | 0.138 |
| Race | 0.041 | 0.566 | 0.053 | 0.092 | 0.046 | 0.267 |
| Period Status | 0.034 | 0.931 | 0.046 | 0.298 | 0.044 | 0.380 |
|  |  |  |  |  |  |  |
| Weighted Unifrac |  |  |  |  |  |  |
| BMI | 0.028 | 0.194 | 0.036 | 0.255 | 0.031 | 0.098 |
| Age | **0.043** | **0.045** | 0.003 | 0.844 | 0.032 | 0.093 |
| Race | 0.037 | 0.554 | 0.026 | 0.611 | 0.035 | 0.537 |
| Period Status | 0.032 | 0.647 | 0.029 | 0.577 | 0.042 | 0.327 |
|  |  |  |  |  |  |  |
| Unweighted Unifrac |  |  |  |  |  |  |
| BMI | 0.031 | 0.061 | **0.049** | **0.029** | 0.025 | 0.185 |
| Age | 0.025 | 0.202 | 0.011 | 0.974 | 0.027 | 0.106 |
| Race | 0.042 | 0.387 | 0.049 | 0.316 | 0.051 | 0.093 |
| Period Status | 0.036 | 0.680 | 0.035 | 0.776 | 0.042 | 0.309 |
