# Supplementary figures and images for: Comparison of gut microbiome composition in colonic biopsies, endoscopically-collected and at-home-collected stool samples
Source: Front Microbiol. 2023 Jun 1;14:1148097. doi: 10.3389/fmicb.2023.1148097 (PMC10264612; doi:10.3389/fmicb.2023.1148097)

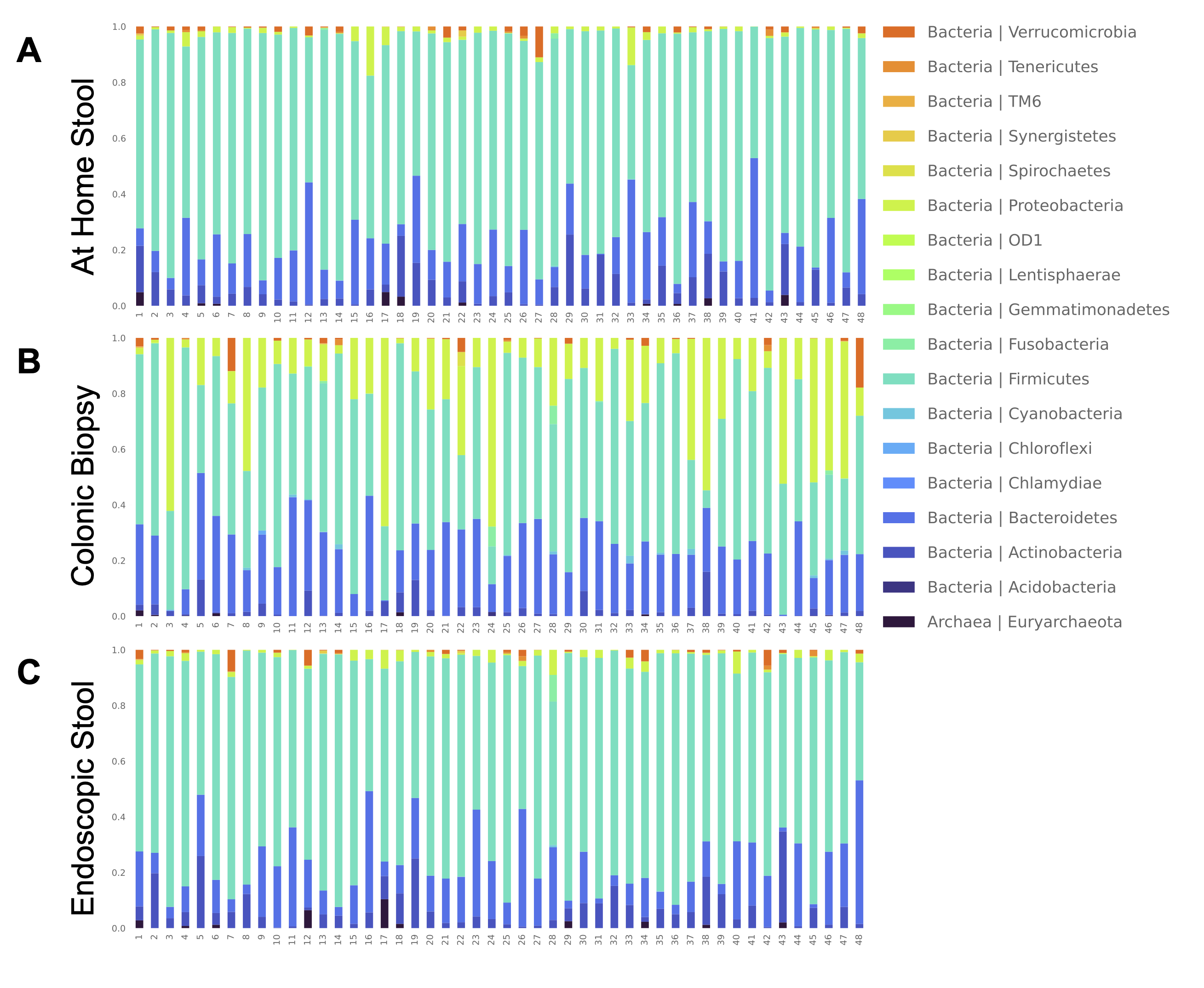

Supplement: Supplementary file 3 [file Image_1.jpg]

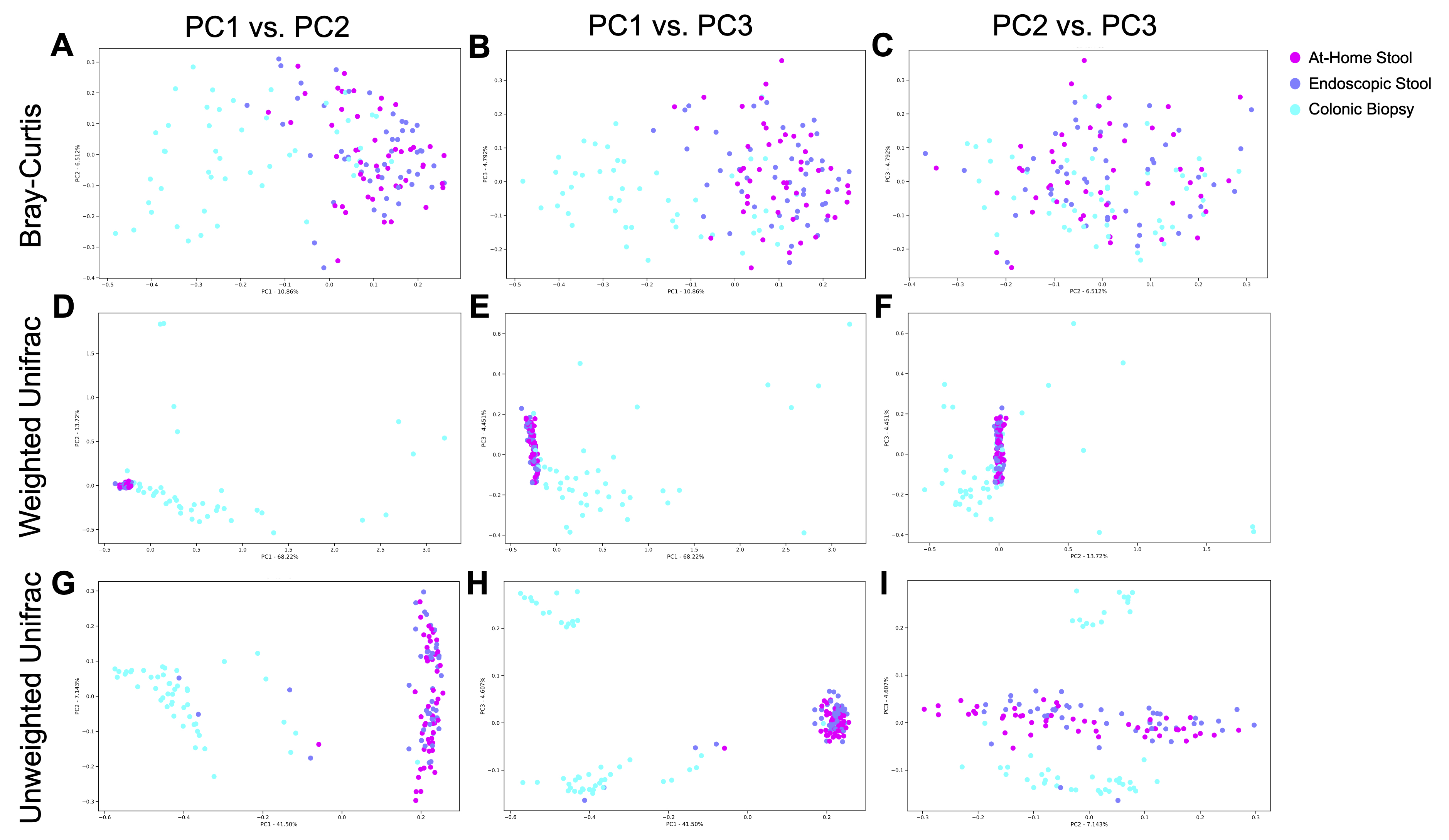

Supplement: Supplementary file 4 [file Image_2.jpg]

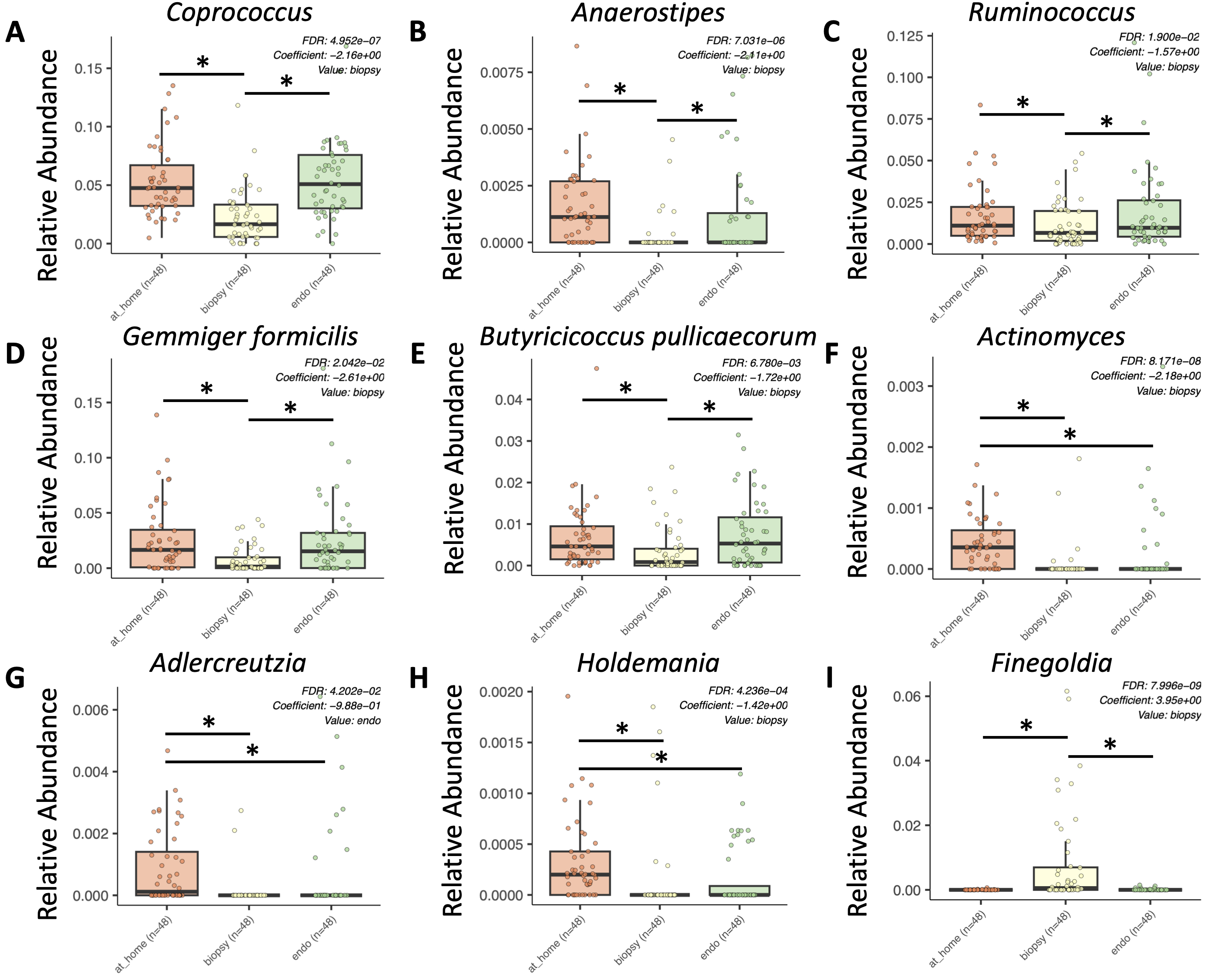

Supplement: Supplementary file 5 [file Image_3.jpg]
